# Nitrogen Interstitial Alloying of CoCrFeMnNi High Entropy Alloy through Reactive Powder Milling

**DOI:** 10.3390/e21040363

**Published:** 2019-04-04

**Authors:** Igor Moravcik, Jan Cizek, Larissa de Almeida Gouvea, Jan Cupera, Ivan Guban, Ivo Dlouhy

**Affiliations:** 1 Institute of Materials Science and Engineering, NETME Centre, Brno University of Technology, Technicka 2896/2, 616 69 Brno, Czech Republic; 2 Institute of Plasma Physics, The Czech Academy of Sciences, Za Slovankou 1782/3, 182 00 Prague 8, Czech Republic

**Keywords:** metallurgy, interstitial, microstructure, powder technology, multi-principal element alloys

## Abstract

The present work is focused on the synthesis of CoCrFeMnNi high entropy alloy (HEA) interstitially alloyed with nitrogen via powder metallurgy routes. Using a simple method, nitrogen was introduced to the HEA from the protective N_2_ gas atmosphere during mechanical alloying (MA) processing. The lattice parameter and amount of nitrogen in HEA were observed to be linearly proportional to the milling duration. The limited solubility of nitrogen in the main face centered cubic (FCC) phase resulted in the in-situ formation of nitrides and, accordingly, significant increase in the hardness values. It has been shown that fabrication of such nitrogen-doped HEA bulk materials can be conveniently achieved by a simple combination of MA + spark plasma sintering processes, without the need for adding nitrogen from other sources.

## 1. Introduction

The need for safety critical application materials with improved combination of high strength, low density, and high fracture resistance has been stimulating research efforts for decades [1]. Lately, high entropy alloys (HEA) consisting of several substitutional elements at near-equiatomic ratios, as well as composites derived from them have been developed [2,3,4]. These deviated from the established, single element-based alloy designs and possessed a combination of promising properties. It is believed that such properties are mostly generated by an extreme substitutional strengthening, arising from the presence of multiple elements within a single solid solution lattice, thereby effectively increasing the effective lattice friction stress [5,6]. Recently, it has been proven that the introduction of interstitial elements to HEA lattices promoted further significant strengthening. Importantly, this is done without sacrificing ductility in the process, thus further pushing the eminent borders of the strength-ductility trade-off [7,8,9,10]. While most attempts benefited from the effects of interstitial carbon or boron, to date, only one study has been recorded to successfully apply nitrogen alloying [11]. This is rather surprising as nitrogen is one of the most important interstitial alloying elements in austenitic stainless steels and CoCr bio-compatible alloys, having a more pronounced effect compared to even carbon or boron, which are more susceptible to the formation of brittle phases [12,13,14]. Partially, the lack of high nitrogen-doped HEA fabricated by traditional metallurgy routes can be explained by their complicated production, as a dissolution of high concentrations of nitrogen in the molten metal requires the use of a high pressure metallurgy [15]. In our study, the interstitial nitrogen-doped HEA produced by a novel manufacturing route is presented. In the production, an economical method of introducing interstitial nitrogen from gas atmosphere during the reactive powder milling process has been utilized. A simple change of the milling atmosphere from Ar to N_2_ gas resulted in the increase in hardness values by 28%.

## 2. Materials and Methods

A set of CoCrFeMnNi (in equimolar proportions) HEA powders were prepared by ball milling of elemental powders with purity over 99.5% and average particle sizes approximately 45 µm. The powders were sealed into a steel milling bowl containing 15 mm diameter milling balls with the ball-to-powder weight ratio (BPR) of 10:1. The used milling speed was 300 revolutions-per-minute for all powders. Total milling durations of 12, 16, and 24 h with N_2_ atmosphere and 16, 20, and 24 h with Ar gas as a reference atmosphere have been performed, without the use of any process control agent. Every 30 min, the milling was stopped for 15 min to prevent overheating of the milling bowl (the provided milling times denote total milling time only, excluding the stop time periods). At the end of every dry milling duration, all powders were additionally wet milled for 15 min in ethanol and subsequently dried in an air oven at 60 °C to increase powder yield. The milling conditions were selected to evaluate the influence of milling time and environment on the chemical composition and hardness of the powders, and subsequently spark plasma sintering (SPS)-consolidated final bulks. To explore an alternative route for nitrogen incorporation, another powder was milled for 24 h under Ar gas atmosphere, this time with added Cr_2_N powder particles. The amount of added Cr_2_N corresponded to 2 at % of nitrogen (0.48 wt %) in the alloy. All milled powders were then consolidated by spark plasma sintering (SPS; Thermal Technology LSS 10-4) in a 20 mm graphite die. A sintering temperature of 1150 °C with 30 MPa pressure and 8 min holding time were used. Graphite foils with applied BN coating were placed between the powders and the die walls to prevent contamination. The milled powders and compacted bulk materials were prepared for microstructural observations using standard metallographic grinding and polishing methods, with last step performed using mechano-chemical polishing using colloidal silica (Struers OPS). For the analyses, SEM (ZEISS Ultra Plus, Carl Zeiss AG, Oberkochen, Germany) equipped with energy dispersive microanalysis (EDS) and electron backscattered diffraction (EBSD) detectors was used. XRD (Philips X´Pert, 40 kV, Co Kα radiation at λ = 1.7903 Å, 2θ = 30–120°, (Philips, Eindhoven, The Netherlands) was used to observe phase compositions at the individual processing steps. The chemical composition of the powders (including the nitrogen concentrations) was determined using LECO THC-600 spectrometer (LECO co., St. Joseph, MI, USA). Vickers micro-hardness measurement of the SPS-ed bulks was carried out using LECO LM 247AT microhardness tester (LECO co., St. Joseph, MI, USA) at a300 g load force and 10 s holding time. THERMOCALC software version 2018 (Thermo-Calc Software AB, Solna, Sweden) was used to calculate the phase composition prediction. Unfortunately, the currently developed HEA database (TCHEA1) could not be used as it does not involve interstitial elements, and its accuracy was questioned [16]. As such, THERMOCALC calculations were realized using an Ni-based alloy database (TCNI9) instead.

## 3. Results and Discussion

**Figure 1:**
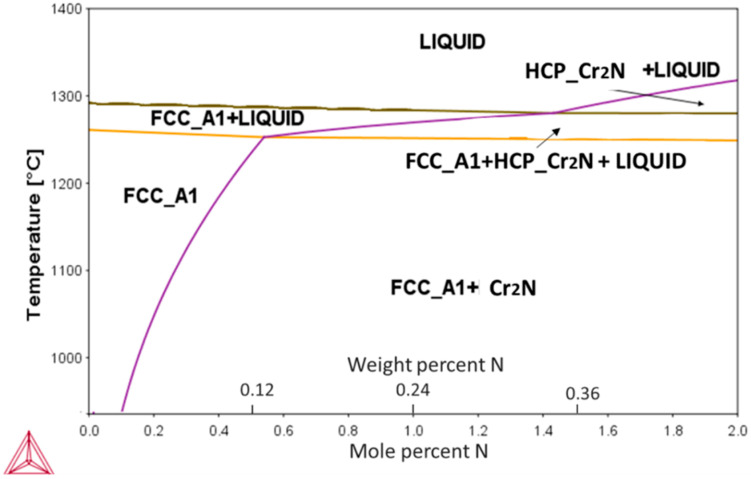
Calculated pseudo-binary CoCrFeMnNi-N phase diagram showing solubility changes of N in the alloy’s main FCC phase with temperature. Formation of Cr_2_N phase can be expected for concentrations exceeding 0.1 at %.

The calculated pseudo-binary phase diagram of an N-alloyed CoCrFeMnNi alloy is presented in Figure 1. Among the present elements, Cr had the highest affinity towards nitrogen, resulting in the appearance of a Cr_2_N phase during the eutectic reaction. The highest solubility of N was ~0.5 at % (0.12 wt %) at the eutectic temperature of ~1280 °C, decreasing rapidly with decreasing temperatures thereafter.

**Figure 2:**
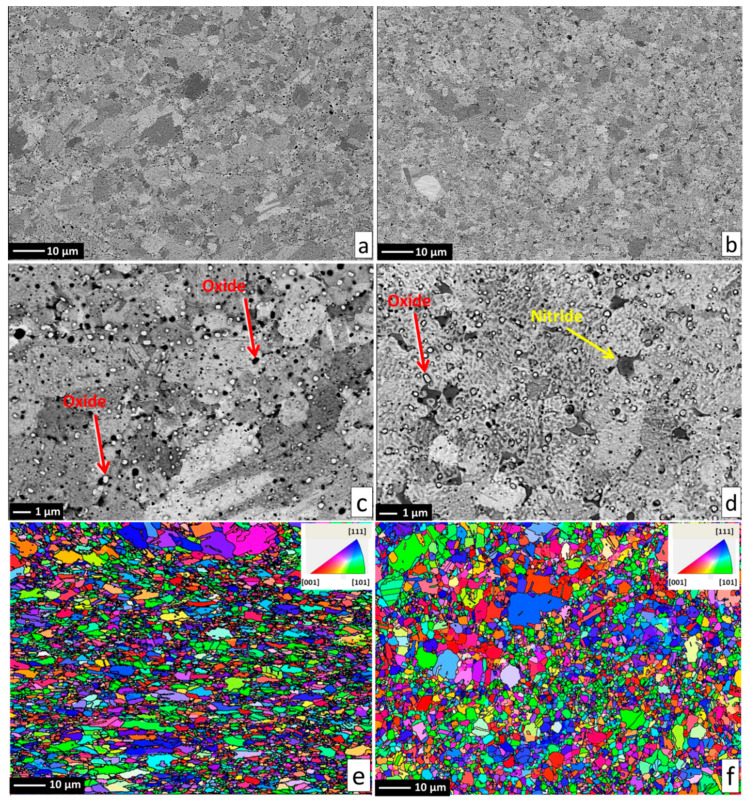
The microstructure and EBSD grain orientation maps of the produced alloys, with reference direction perpendicular to the SPS compaction direction. Pure CoCrFeMnNi alloy presented on the left hand side in (**a**,**c**,**e**); CoCrFeMnNiN alloy presented on right hand side in (**b**,**d**,**f**) with the formed Cr_2_N phase denoted by yellow arrow in (**b**). Inevitable oxide particles are highlighted by red arrows in (**c**,**d**).

**Figure 3:**
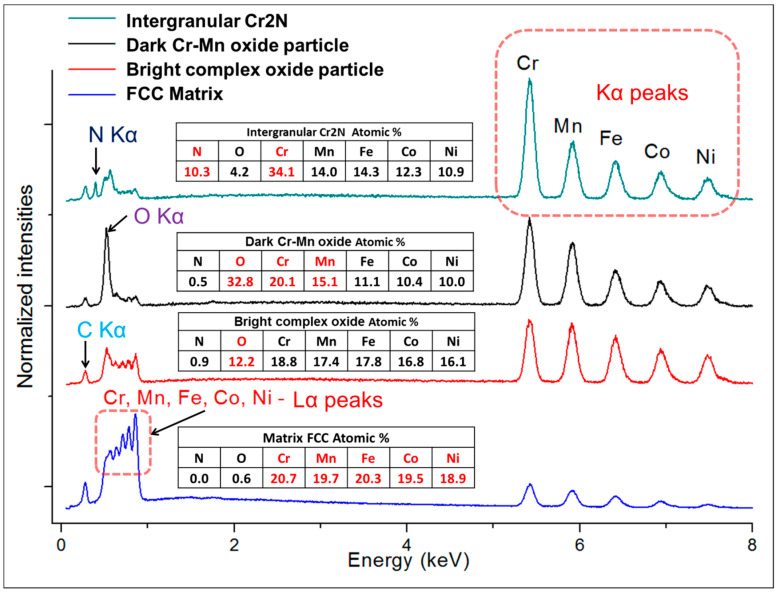
The representative point EDS spectra taken from the respective phases present in the microstructures of SPS-ed bulks of CoCrFeMnNi and CoCrFeMnNiN: matrix, white-contrast Cr- and Mn-rich oxides, dark-contrast oxides, and the intergranular Cr_2_N grains. Note that considering the interaction volume’s size of the incident electron beam and the respective size of the oxide particles and nitride grains (generally below 1μm), the results are not accurate in terms of exact quantification. The carbon peaks present in all patterns are a consequence of the sample preparation.

Alloy microstructures prepared using SPS from the powders subjected to 24 h of milling in Ar and N_2_ atmospheres are presented in Figure 2a–d, respectively. In all cases, fully-dense bulk samples were obtained, justifying the selection of SPS parameters. The microstructure of pure CoCrFeMnNi alloy is composed of single FCC phase grains and inevitable oxide inclusions, visible as fine dark and bright particle dispersion. The difference in the oxide particle contrast is given by relative differences in their chemical composition: while the dark particles shown in Figure 2c,d are mostly composed of chromium and manganese oxides, the white particles are also oxides. However, their spectra indicate a significantly lower oxygen content (12 at % vs. 33 at %) and higher quantities of the three heavier metallic elements (Fe, Co, Ni; see the spectra and respective compositions provided in Figure 3). Both decreased oxide content and the higher presence of the three heavier elements triggered the visible color difference. It should be noted that the EDS method is not perfectly suited for determining light elements such as N or O, and the presented results are therefore not meant to represent an exact quantification. The dark sheath visible around the white-contrast oxides in Figure 2c particles is an effect caused by the used mechano-chemical polishing (hard and resistant Cr- and Mn-rich oxide particles are protruding from the matrix that was partially etched away). Importantly, the CoCrFeMnNi-N bulk alloy produced from powders with 0.2 wt % of N contained additional, intergranular Cr- and N-rich phases (confirmed by EDS, see Figure 3). This phase corresponds to a Cr_2_N nitride phase from the calculated prediction in Figure 1. As this phase was not observed (XRD, EDS) in the powder prior to the SPS process, its presence is connected to the decreasing solubility of nitrogen in the FCC matrix upon slow cooling after sintering. This provided sufficient time for the nucleation of Cr_2_N grains at the main FCC phase grain boundaries (pathways to elemental diffusion). The EBSD orientation maps (Figure 2e,f) demonstrated that after the SPS process, microstructures were fully recovered, indicated by a single-color (single crystallographic orientation) representation of each grain (should plastic strain be still present after the SPS, different shades of the individual colors would be visible within each grain). Pure CoCrFeMnNi and CoCrFeMnNi-N alloys possessed almost identical average grain size of the main FCC phase, quantified using the EBSD method to be 3.78 ± 1.16 μm and 3.53 ± 1.05 μm, respectively. The identical average grain sizes (considering given uncertainty) despite the presence of intergranular Cr_2_N in the CoCrFeMnNi-N bulk alloy further supports the hypothesis on the nitride precipitation only during the cooling period from the SPS consolidation temperatures. In theory, an earlier formation of nitride (that is, during the powder milling or first half of the SPS sintering process) would have actually prevented grain growth of the CoCrFeMnNi-N material. This would necessarily have resulted in a large difference in the average grain sizes between both alloys. However, this was not confirmed experimentally (note the two values of grain size are not statistically different considering the scatter values), which points to the fact that nitrides formed during the final SPS cooling phase only.

**Figure 4:**
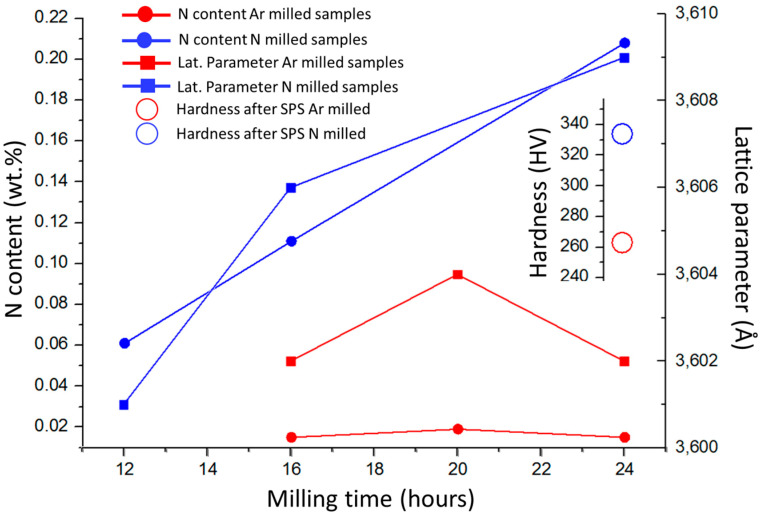
The influence of milling time and reactive atmosphere on the total measured nitrogen content in the powders, corresponding lattice parameters of the powders, and resulting hardness of the SPS compacted bulks after sintering; Ar and N series refer to powders milled under Ar and N atmospheres, respectively.

Influences of powder milling time and used atmosphere on the chemical composition (nitrogen content), main FCC phase lattice parameter of the produced powders, and hardness of the bulk samples after consolidation, are presented in Figure 4. Milling in nitrogen atmosphere caused adsorption of N atoms at the powder particle surfaces, and its subsequent dissolution into the particles, which resulted in a significant increase in nitrogen concentration within the compacts. The performed XRD analysis revealed that all the powder materials and the bulks were composed of a single FCC phase. The lattice parameters of the phase were found by increasing with the increasing N concentrations in the powders. The observation is in good agreement with previous results stating that lattice parameters increase by interstitial alloying [7,17]. Unfortunately, volume fraction of the Cr_2_N and minor oxide phases was insufficient to enable their detection by XRD. The corresponding lattice parameters of SPS compacted bulks of CoCrFeMnNi and CoCrFeMnNi-N alloys were 3.600 and 3.606 Å, respectively (not displayed in Figure 4). Despite Cr_2_N phase formation, this result would suggest that a certain content of nitrogen remained dissolved in the FCC solid solution of the CoCrFeMnNi-N bulk. Figure 4 shows that the introduction of N resulted in the increase of SPS-ed materials’ hardness by ~28% compared to the unalloyed counterparts. Such increase is mostly induced by Cr_2_N phase formation, i.e., secondary phase dispersion strengthening. The (small) difference in lattice parameters of bulk CoCrFeMnNi and CoCrFeMnNi-N alloys further suggests that the hardness increase could have been partially caused by interstitial solid solution strengthening, too [18,19]. Comparing nitrogen bulks alloyed from milling in N_2_ gas atmosphere with the corresponding alloy produced (using identical parameters) under Ar atmosphere by adding Cr_2_N powder, it was shown that the latter exhibited ~4% lower hardness values. Such result is surprising, especially considering the total introduced nitrogen concentrations (0.2 wt % for gas-introduced N vs. 0.38 wt % for Cr_2_N particles-introduced N present in the powders after milling). This is most likely a consequence of the significantly smaller sizes of (in-situ formed) Cr_2_N particles in the gas-alloyed bulk, triggering higher strengthening efficiency, despite its lower total volume. It is interesting to point out that the concentration of N introduced to powders prior to milling in the form of added Cr_2_N particles (0.48 wt %) is different from that measured in the alloyed powders after the milling procedure (0.38 wt %). The difference could be attributed to Cr_2_N phase decomposition during milling. In summary, the introduction of nitrogen is achieved easily by simple milling in the N_2_ gas atmosphere after relatively short milling durations already. Such milling process does not require any additional steps.

## 4. Conclusions

In this work, we focus on the production of interstitial CoCrFeMnNi-N high entropy alloys via simple and economical method of reactive milling of elemental powders in nitrogen atmosphere. The main results of the study can be summarized as follows:

- As opposed to metallurgical routes, nitrogen can be easily introduced into CoCrFeMnNi alloy powders by reactive mechanical milling in N_2_ atmosphere.
- Nitrogen content in the final products can be adjusted by altering the milling duration.
- Due to a limited solubility of nitrogen in the FCC solid solution phase, formation of additional Cr_2_N nitride phases upon sintering is observed at higher nitrogen concentrations
- The introduction of nitrogen into the CoCrFeMnNi HEA results in improved hardness by combining the second phase dispersion and solid solution interstitial strengthening effects.
